# The Use of the Acoustic Emission Method to Identify Crack Growth in 40CrMo Steel

**DOI:** 10.3390/ma12132140

**Published:** 2019-07-03

**Authors:** Aleksandra Krampikowska, Robert Pała, Ihor Dzioba, Grzegorz Świt

**Affiliations:** 1 Department of Strength of Materials, Concrete and Bridge Structures, Kielce University of Technology, Al. 1000-lecia PP 7, 25-314 Kielce, Poland; 2 Faculty of Mechatronics and Mechanical Engineering, Department of Machine Design, Kielce University of Technology, Al. 1000-lecia PP 7, 25-314 Kielce, Poland

**Keywords:** pattern recognition, acoustic emission, Structural Health Monitoring, brittle fracture, diagnostics

## Abstract

The article presents the application of the acoustic emission (AE) technique for detecting crack initiation and examining the crack growth process in steel used in engineering structures. The tests were carried out on 40CrMo steel specimens with a single edge notch in bending (SENB). In the tests crack opening displacement, force parameter, and potential drop signal were measured. The fracture mechanism under loading was classified as brittle. Accurate AE investigations of the cracking process and SEM observations of the fracture surfaces helped to determine that the cracking process is a more complex phenomenon than the commonly understood brittle fracture. The AE signals showed that the frequency range in the initial stage of crack development and in the further crack growth stages vary. Based on the analysis of parameters and frequencies of AE signals, it was found that the process of apparently brittle fracture begins and ends according to the mechanisms characteristic of ductile crack growth. The work focuses on the comparison of selected parameters of AE signals recorded in the pre-initiation phase and during the growth of brittle fracture cracking.

## 1. Introduction

Diagnosing and monitoring the condition of structures is an important and very topical area in construction. Aging infrastructure and increasing service load of engineering structures are the main drivers of fast-progressing research in the new interdisciplinary field of knowledge called structural health monitoring (SHM), which is closely related to the durability and safe operation over the life of the structural elements.

The load is a typical random load that is difficult to model with currently known fatigue calculation procedures, and can be modelled only for steady loads with low-level amplitudes. As a result, the fatigue life calculation models for steel structures fail to provide accurate information about the risk of fatigue failure. Where calculation methods provide only limited information, the existing structures are subjected to tests. Fatigue damage testing is carried out during inspection. Cracks, which occur for a number of reasons, are detected and, in the case of fatigue cracks, the rate of their growth is assessed.

Depending on the operating conditions, the loading and materials used, the failure takes place according to a ductile or brittle fracture mechanism.

Ductile cracking usually occurs in steels and is preceded by significant plastic deformation that manifests itself long before the failure of the element, thus allowing preventive measures to be undertaken. The brittle fracture mechanism is much more dangerous.

Brittle cracking proceeds without visible deformation of the element and occurs almost immediately. The occurrence of brittle fracture can be expected mainly in high-strength structural steels or in low-temperature operation and overloading of the element with simultaneous increase in the load speed. Welded joints are also exposed to brittle fracture due to their inhomogeneous microstructure, welding defects and residual stresses. The presence of these factors together with the impact of fatigue loads and corrosive environment leads to the degradation of the material and development of micro-cracks, resulting in the brittle failure of steel.

Leading research centers worldwide have devoted a great deal of attention to the development of methods for assessing the condition of structural elements and preventing their failure. As a result, many methods for evaluation of component durability [1,2,3,4,5] are currently in wide use. These methods are based on aversion to change assuming prior knowledge of the element microstructure, load history, and in-service conditions. However, more accurate results will be obtained when as much information as possible can be collected on in-service parameters and material.

Hence, the modelling of bearing capacity and durability of a structure under real operating conditions requires that:

- real operational loads are defined;
- the material model is defined, in particular welded joints material that changes over time under the influence of operating conditions;
- the load around the defect after its initiation and during development (redistribution of stresses) is determined; and
- the interaction of various damage types is identified.

As determining the factors above is very difficult, if not impossible, most analyses are characterized a high degree of conservatism, which significantly reduces assessment accuracy. Appropriate solution should include developing an NDT-based monitoring system able to signal the structural safety risk.

Finding and determining the “destructive characterization” of all hot spots in large-sized structures using the NDT methods is nearly impossible. In steel structures, the volume of “hot spot” is of the order of cubic centimeters, with dimensions of the structure reaching tens and more meters. In our opinion, the solution to the problems of diagnosing engineering structures is the use of continuous, long-term monitoring using the passive NDT methods. One of them is the acoustic emission (AE) method [4,5,6,7,8,9,10,11,12,13,14].

The paper presents a proposal for the use of acoustic emission for the diagnosis of steel structures vulnerable to brittle fracture.

The choice of acoustic emission as the research method was determined by its advantages in relation to other non-destructive methods. These are:

- locating the faults that are undetectable with conventional methods;
- recording only active damage, i.e., the defect growth as it occurs;
- continuous monitoring of structures while in service or during load tests, with continuous data recording;
- detecting all types of damage, whereas most other methods focus on particular defects;
- characterising the rate of damage development during the operation of the structure; and
- enabling characterization of AE signal sources.

The main task of the measurement system consisting of an AE processor is to detect, record, filter and analyze the signals generated by AE sources. 

Each destructive process is a source of acoustic emission. The source is described by the parameters of the recorded AE signal. The values of the parameters are used to classify the signals (and, thus, damage processes). The similarity of signals is used to attribute particular signals to the defects caused by specific damage processes, and then by applying statistical grouping methods to identify the existing defects.

For the statistical methods used in identification, an important issue is the optimal selection of recorded 13 AE parameters (counts, counts to peak, amplitude, RMS—root means square voltage, ASL—average signal level, energy, absolute energy, signal strength, rise time, duration, initiation frequency, average frequency, reverberation frequency). The parameters must be characterized by low mutual correlation. A set of diagnostic variables must describe the most important aspects of the studied phenomenon [15,16,17,18]. Techniques such as hierarchical and non-hierarchical clustering methods and Kohonen’s neural networks are used to build the reference signals data base for identification of destructive processes in steel structures (IPDKS) [4,5].

This paper uses unsupervised pattern recognition methods to characterize different AE activities corresponding to different fracture mechanisms. A sequential feature selection method based on a k-means clustering algorithm is used to achieve high classification accuracy. Fatigue damage propagation represents the main failure factor. To study the contributions of different types of damage at progressive fatigue stages, a tool with the ability to detect the damage initiation and to monitor failure progress online is needed. Acoustic emission (AE) testing has become a recognized suitable and effective non-destructive technique to investigate and evaluate failure processes in different structural components. The main advantage of AE over other condition monitoring techniques is that detected AE signals can be used to characterize the different damage mechanisms. The approach of using parameter distribution has the advantage of real-time damage detection, but can also lead to false conclusions due to noise effects in the AE signals [18]. This is especially true for materials that are working under the fatigue conditions which are usually present in damage mechanism interactions. Therefore, AE techniques are needed to account for more intricate wave propagation features caused by the anisotropic nature of materials and to enable the identification of a large variety of failure modes. It is now possible to detect and capture very large numbers of AE signals, driving a trend for seeking computationally complex algorithms, such as pattern recognition, to determine the onset of significant AE. For a real structure it is not possible to provide a set of training patterns belonging to multiple damage mechanisms, which thus makes the use of unsupervised pattern recognition techniques more appropriate for these studies [18]. 

The first step of developing an unsupervised pattern recognition process is to classify signals into groups based on similarities. This process involves statistical effects, and the key point of successful feature selection to construct fine classification accuracy. A paper by Doan et al. [16] presented a feature selection method that introduced a sequential method based on the Gustafson–Kessel clustering algorithm. In these the method, the subset of features is selected by minimizing the Davies-Bouldin (DB) index, which is a metric for the evaluation of classification algorithms. The signals are classified into four groups by comparing their features and deciding upon their similarity. 

The assignment of the clustering results to the fracture mechanisms is achieved by a detailed analysis of the physical meaning of the data [19,20,21,22]. This is the first time that the pattern recognition technique has been applied to a database acquired from such a complex structure in a fatigue testing environment [16]. The applied feature selection algorithm proves to be a powerful tool providing relevant clustering when used together with a k-means algorithm. When a crack occurs in the material, it results in a rapid release of energy, transmitting in the form of an elastic wave, namely acoustic emission (AE). The AE-based detection method has been intensively used also in non-destructive assessments of cracks in papers [23,24,25]. Additionally, Rabiei and Modarres revealed a log-linear relationship between the AE features and crack growth rate, and presented an end-to-end approach for structural health management [10]. Qu et al. presented a comparative study of the damage level diagnostics of gearbox tooth using AE and vibration measurements; the results indicated that vibration signals were easily affected by mechanical resonance, while the AE signals showed a more stable performance [26]. Zhang et al. have studied defect detection of rails using AE and wavelet transform at a high speed [27]. Li and al. created a template library of cracking sounds and designed a detection device using voiceprint recognition with an accuracy of 77% [28]. Hase et al. combined the Hurst exponent and the neural network to develop a crack detection algorithm of carbide anvils [27]. In the above methods, the AE sensors are usually attached to the surface of the monitoring object, what makes measurement difficult.

A more practical crack identification and detection method is still lacking. Aiming to improve recognition accuracy and generalization, in papers [29,30,31] a novel crack identification method based on acoustic emission and pattern recognition is proposed. In these methods, the sound pulses from cracks are firstly separated from the original signal by pre-processing. The high-dimensional features are reduced adaptively by using principal component analysis (PCA). The algorithm combines a k-nearest neighbor (kNN) classifier with a support vector machine (SVM) to refine the classification outcome. While debris monitoring does not require any electronics, it is simple to interpret and has excellent sensitivity to wear-related failure; this method is insufficient to non-benign cracks as no debris is produced. Acoustic emission (AE) and vibration signals have more quantitative results to detect the earliest stage of damage in rotating machinery. AE and vibration methods are based on recording transient signals in two different frequency spectrums. While vibration method is based on features that are extracted from time and frequency domain signals recorded by low frequency accelerometers in order to assess the changes in vibrational properties as related to the damage [29,30,31], the AE method is based on detecting propagating elastic waves released from active flaws. Once transient signals are collected, signal processing methods, such as wavelet decomposition [11], empirical mode decomposition [11], and multivariate pattern recognition [20], are applied. Typical parameters extracted from the transient signals are root mean square value, frequency domain characteristics, energy, spectral kurtosis, and peak-to-peak vibration level. Due to the difference in the frequency bandwidth, the AE method is more sensitive to microcracks as compared to the vibration method. Typical AE data acquisition approach is based on threshold: an AE signal is detected when the signal level is above threshold. As crack growth is a stochastic process, it is considered that while some data will be lost due to the idle time of data acquisition system between waveform recording intervals, crack information will be stochastically detected. However, it is important to identify how to analyze long-duration signals in order to reduce the influence of background noise from the extracted features. In studies, the AE signals, which accompanying of the fatigue crack growth is important obtained from the scaled laboratory experiments. Acoustic emission (AE) is a health monitoring approach which acts as a passive receiver to record internal activities in structures. This method is capable of continuous monitoring, which is not the case with most traditional methods. In addition, AE sensors are very sensitive and can capture signals due to micro-scale defect formations coming from the internal regions of structures rather than only those at the surface [18,23]. An unsupervised and supervised pattern recognition algorithm was employed to classify the AE signals. Different damage mechanisms for specimens during cracking were identified using reference database AE signals created from signals registered in the tests described below.

## 2. Materials and Methods 

**Figure 1:**
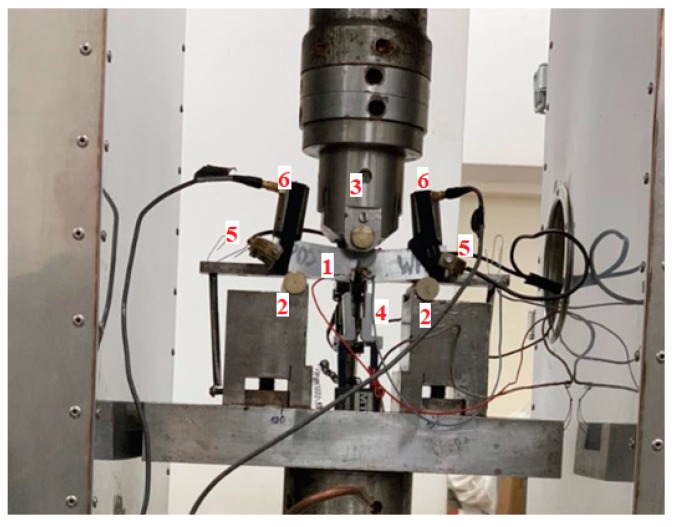
The view of the SENB specimen with AE sensors (Zwick-100, ZwickRoel Ulm, Germany) during test: 1—a sample, 2—support, 3—load cylinder, 4—COD extensometer, 5—AE sensors—100–1200 kHz, 6—AE sensors—30–80 kHz.

**Figure 2:**
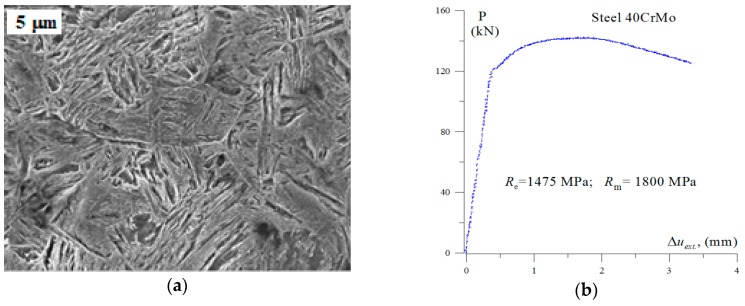
(**a**) Microstructure of tempered martensite of 40CrMo steel; and (**b**) loading plot of a cylindrical specimen in uniaxial tensile test.

The tests discussed below were carried out using Zwick-100 testing machine (ZwickRoel Ulm, Germany ) on SENB (Single Edge Notch in Bending) specimens (Figure 1) made of 40CrMo steel. The specimens with dimensions 12.5 mm × 25 mm × 110 mm were subjected to heating at 850 °C (15 min), quenching in oil, tempering at 250 °C (3 h) and cooling in oil. As a result, a material with a tempered martensite microstructure was obtained (Figure 2a). The signals of loading, specimen deflection and acoustic emission (AE) were recorded during testing.

SENB type specimens with a total fracture length, which includes a notch + previously fatigue crack, equal to about 0.5∙*a*/*W* are recommended by the ASTM and PN-EN standards [32,33,34,35] and commonly used to determine fracture toughness characteristics—critical values of the stress intensity factor (SIF) *K*_IC_ or the critical *J-integral*, *J*_IC_. If during the entire process of loading the SENB specimen in the net-section area before the crack tip there is a definite predominance of the linear-elastic nature of the stress and strain fields before the crack tip are described by SIF and the fracture toughness is characterized by the critical value of *K*_IC_. The condition allowing the use of SIF is to limit the size of the yielding before the crack tip: *r*_p_ ≤ 0.01∙*a*, where for a plane strain *r*_p_ = (1/6*π*)∙(*K*_I_/*σ*_y_)^2^. If the plastic zone is larger, it means that the material in front of the fracture tip is elastic-plastic and for the description of mechanical fields an integral *J* must be used, and fracture toughness is represented by the critical integral *J* – *J*_IC_ [36,37].

For determining material strength characteristics, cylindrical specimens 10 mm in diameter were prepared with the measuring section of 50 mm. For determining the critical value of fracture toughness, *K*_IC_, the specimens with dimensions 12.5 mm × 25 mm × 110 mm with one-sided notch and crack fatigue (SENB) with a total length of about 12.5 mm were made. Figure 2b shows an example plot of loading a cylindrical specimen. On the basis of the graphs, strength characteristics of 40CrMo steel were determined: *R*_e_ = *σ*_y_ = 1475 MPa; *R*_m_ = *σ*_uts_ = 1800 MPa; *E* = 205 GPa. During the loading of the SENB specimen, it was determined that the crack growth process takes place according to the brittle fracture mechanism. All standard conditions were met. The obtained critical value of fracture toughness is a material characteristic and is equal to *K*_IC_ = 39 MPa∙m^1/2^.

The reference signal database in the IPDKS method for steel structures was developed using the Fuzzy k-means algorithm. The k-means algorithm belongs to a group of non-hierarchical clustering approaches. Its essence lies in the random choice of initial centres. In each iteration further approximations of the patterns are searched for and enumerated using the given methods. Depending on the assumptions made, the reference element may be one of the elements of the *X* population or belong to a certain universe *U* *⊇ X*.

In metric spaces, a reference element can be calculated as an arithmetic mean to represent the centre of gravity of the cluster.

In general, the *k-clustering* algorithm input is a set of *X* objects and the expected number of clusters *k*, and the output is the division into subsets *{C_1_, C_2_, …, C_k_}*. Frequently, ***k-clustering*** algorithms belong to the category of optimization algorithms. Optimization algorithms assume that there is a loss function *k: {x | X* *⊆ S} → R +* specified for each *S* subset. The aim is to find a branch due to minimizing the sum of losses described by Equation (1):

(1) $$E_{q}=\overset{k}{\underset{i=1}{\sum }}k\left(C_{i}\right)$$

In order to apply the iterative algorithm, the distance measure used should be determined in the grouping process. Instead of the median, you can use a point that is the resultant point for a given cluster (representation element) calculated, e.g., as a geometric or arithmetic mean. Then we deal with k-means (k-means) algorithms described by Equation (2):

(2) $$k(C_{i})=\sum_{r=1}^{\left|C_{i}\right|}d\left({\bar{x}}^{i},\;x_{r}^{i}\right)$$

where: ${\bar{x}}^{i}$—the arithmetic mean (or geometric) of the cluster.

Doing so means that the centroids search for their correct positions using Equation (3) [4,5]:

(3) $${\underline{\mu }}_{j}=\frac{\sum_{j=1}^{n}P{\left(\omega_{i}{|\underline{x}}_{j}\right)}^{b}{\underline{x}}_{j}}{\sum_{j=1}^{n}P{\left(\omega_{i}|{\underline{x}}_{j}\right)}^{b}},$$

where $P\left(\omega_{i}{|\underline{x}}_{j}\right)$ is the conditional probability of belonging *j*-th element to the i-th group, *b* is the parameter that has to take values other than 1, ${x\;\_}_{j}$ is the *j*-th element.

The probability function is normalized according to Equation (4):

(4) $$\sum P\left(\omega_{i}{|\;x\;\_}_{j}\right)=1,\;where\;j=1,\ldots ,n,$$

The probability of the membership of the element in each of the clusters $P\left(\omega_{i}{|\underline{x}}_{j}\right)$ is calculated from Equation (5):

(5) $$P(\omega_{i}{|\underline{x}}_{j})=\frac{{\left(\frac{1}{d_{ij}}\right)}^{\frac{1}{b-1}}}{\sum_{r=1}^{c}{\left(\frac{1}{d_{rj}}\right)}^{\frac{1}{b-1}}},$$

where $d_{ij}{}^{2}={\left\|{\underline{x}}_{j}-{\underline{\mu }}_{i}\right\|}^{2}$ is the distance of a data point ${\underline{x}}_{j}$ from the center of the group ${\underline{\mu }}_{i}$.

The *k-means* algorithm consists of the following steps:

1. Randomly select initial centroids.
2. Compute the distances between the data points and the cluster centroids.
3. Compute the membership function value of all elements $P\left(\omega_{i}{|\underline{x}}_{j}\right)$.
4. Compute cluster centroids ${\underline{\mu }}_{i}$.
5. If:
  - there are no changes in ${\underline{\mu }}_{i}$ and $P\left(\omega_{i}{|\underline{x}}_{j}\right)$- return ${\mu \;\_}_{1}$ … ${\;\mu \;\_}_{c}$,
  - otherwise go back to Step 2.

When this algorithm is used, the number of clusters is pre-determined. However, the speed of computation compensates for this inconvenience. The reference signals database is created with 13 correlated AE parameters (rise time, durability, counts, counts to peak, energy, RMS, ASL, amplitude, average frequency, initiation frequency, reverberation frequency, absolute energy, signal strength). Reference signals obtained in this way make it possible to identify individual destructive processes in all tested structures. The database can be supplemented as per the diagram above whenever new measurement data are obtained.

The use of the grouping method is particularly important for the identification of fracture mechanisms as it allows for timely response to the possible averse effects.

The study used a 24-channel “µSamos” acoustic emission processor, 40 dB preamplifiers and two resonance sensors with flat characteristics in the 30–80 kHz range and two broadband sensor in the 100–1200 kHz range. The use of four sensors with flat characteristics was aimed at determining the lower and upper discrimination thresholds of the AE signal, while the broadband sensor had the task of controlling whether during the measurement there are no signals generated in other frequency bands.

## 3. Results and Discussion

During the loading of the SENB specimen, the crack growth process takes place according to the brittle fracture mechanism and critical value of fracture toughness is a material characteristic equal to *K*_IC_ = 39 MPa∙m^1/2^. At the critical moment, the length of the plastic zone according to the Irwin model for plane strain equals: 2*r*_p_ = 2(1/6*π*)∙(*K*_IC_/*σ*_y_)^2^ = 0.074 mm. According to Williams’ formulas in front of the plastic zone in the crack plane direction, the level of stress tensor components is *σ*_xx_ = *σ*_yy_ = 1808 MPa, *σ*_zz_ = ν∙(*σ*_xx_ +*σ*_yy_) = 1193 MPa. Directly in the plastic zone the stress components are significantly higher and the stress opening component, *σ*_yy_, can obtain values higher than 3*σ*_y_, i.e., over 4500 MPa [38]. The *σ*_zz_ and *σ*_xx_ components are lower, but also exceed the level of 2*σ*_y_.

High stress levels in front of the crack tip cause material damage processes, cracking development through various mechanisms: brittle fracture on the cleavage planes, brittle intergranular cracking, ductile fracture through nucleation, growth and coalescence of voids. The predominance of any fracture mechanism depends on the stress-strain state in the tested element, on the microstructure type, loading method, the temperature of the environment, and on other effects. Here, the situation was seemingly simple—SENB specimens crack according to the brittle mechanism. The AE method was used to monitor the processes during loading of steel specimens.

**Figure 3:**
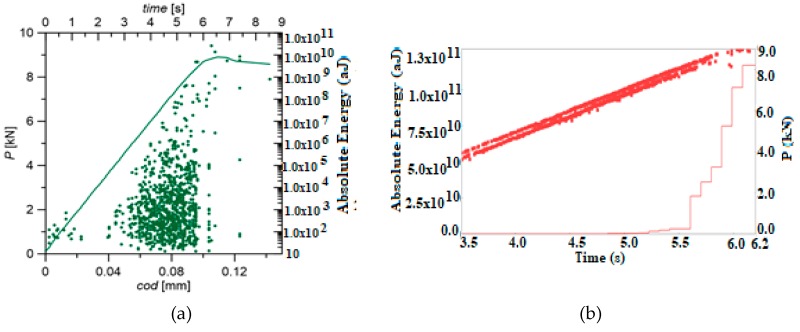
The graph of specimen loading (**a**) with absolute energy of AE signals (aJ) and (**b**) graph cumulative absolute energy vs. load on time from 3.5 to 6.2 s. The graphs show AE parameters without using the clustering method (identification) of destructive processes.

Five specimens were tested. The load plots and the character of AE signals distribution were similar in all tested samples. Figure 3b shows the characteristic loading curve and numerous points of the absolute energy of AE signal and the graph illustrating the cumulative absolute energy vs. load on time from 3.5 to 6.2 s. The graphs show AE parameters without using the clustering method (identification) of destructive processes.

Figure 3a shows the change in the absolute energy parameter (aJ), and the specimen loading as a function of time and COD (crack opening displacement). Acoustic emission signals generated during the study showed an increase in their numbers and increase in the value of absolute energy parameter at COD of 0.045 (mm) and 4.2 kN of load. It can be noticed that the increase in the value of the absolute energy parameter occurs when the cracking process begins (initiation, propagation).

**Figure 4:**
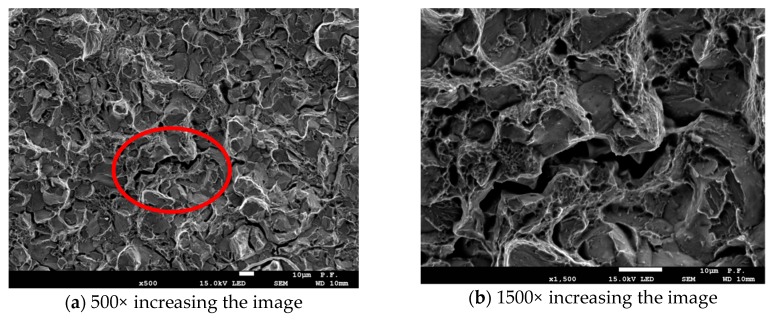
Morphology of the fracture surface the SENB specimen breakthrough: (**a**) general character of fracture surface; and (**b**) details of fracture mechanisms.

During the observations of the fracture surface of the specimens examined using the scanning microscope, three basic mechanisms in the crack development process were identified. The images shown in Figure 4 are presents the same fragment the fracture surface, but at different magnitude. In Figure 4a, at 500×, we can see the general character of brittle fracture surface. In Figure 4b, at 1500×, the details of fracture mechanisms are shown. At the fracture surface, two types of brittle fracture were present: *intercrystallite* cracking and *transcrystallite cleavage*. (Figure 4a,b). There were also areas where cracking developed according to the ductile mechanism, that is, where voids nucleated, grew, and coalesced around the small particles of precipitations (Figure 4b).

Observations of the process of cracking over time shows that at first cracking proceeds according to brittle mechanisms, while cracking due to sudden immediate coalescence of voids is the last stage of crack growth. These observations were confirmed by the analysis of AE signals generated in the tested SENB specimen. However, in order to be able to detect the differences in the processes accompanying the cracking of steel specimens, the signal grouping method must be used. Figure 4a, b shows the selected AE parameters (absolute energy) not analyzed using the clustering methods. It can be noticed that it is difficult to interpret the changes in the work of the tested element. It is impossible to assess the moment of initiation of the crack or its subsequent propagation. It can be said that individual parameters not analyzed by clustering methods do not help in the interpretation of changes in the tested samples. That is why it is so important to use Big Data analysis to create and identify destructive processes on the basis of the pattern recognition methods.

**Figure 5:**
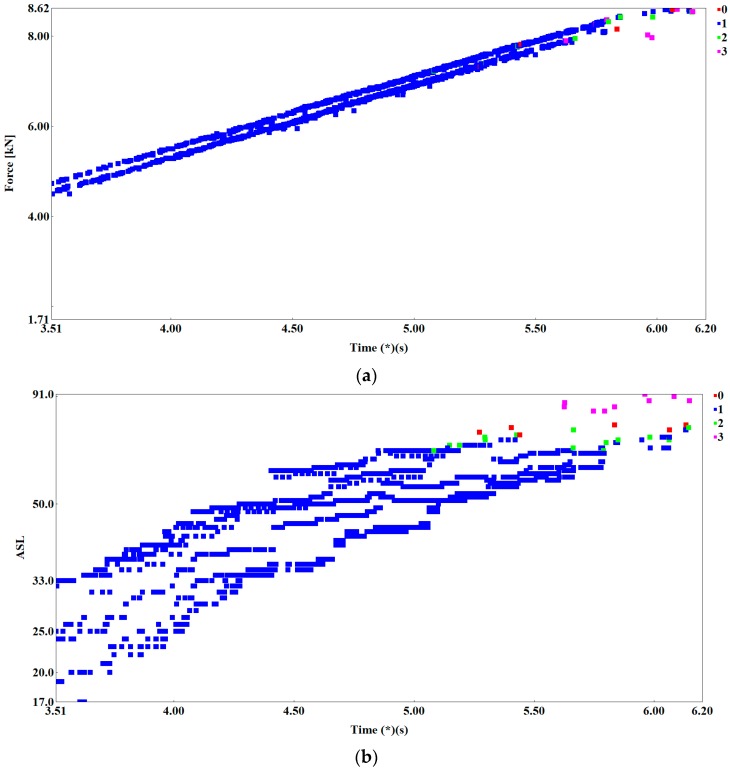

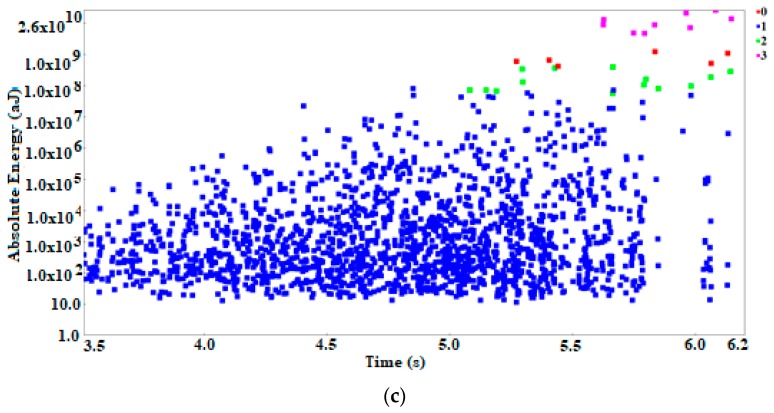
Scatterplots of acoustic emission signals: (**a**) Force (kN) vs. time (s); (**b**) ASL (dB) vs. time (s); and (**c**) absolute energy (a) vs. time (s).

Using one of the grouping methods, namely the fuzzy k-means algorithm, we developed the reference signal data base describing the processes of brittle fracture during destructive tests of steel structures. The AE signals generated and recorded in the crack initiation and development area were subjected to a grouping analysis using the NOESIS 5.8 program, which allowed to separate four basic classes. Figure 5a–c present the effects of using method of grouping signals—a non-hierarchical k-means method to identify mechanisms accompanying the brittle fracture.

**Figure 6:**
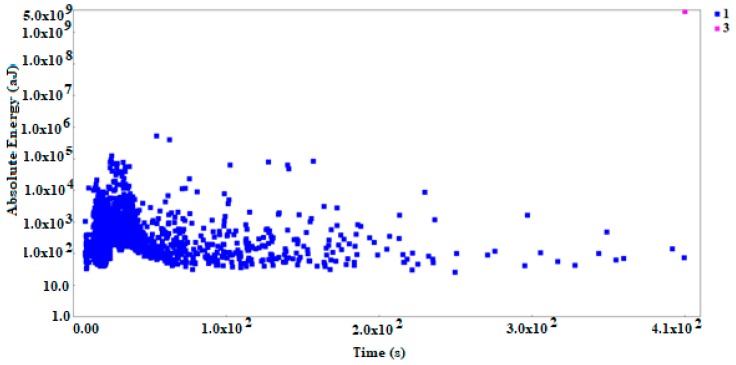
Scatterplot: Absolute energy (aJ) vs. time (s) for uniaxial tested specimen.

Analyzing the division of AE signals into the classes marked in Figure 5 with colors and figures and using photographs (Figure 4) from the scanning microscope, it can be noticed that cracking due to sudden immediate combination of the voids, breaking bridges between them are pink signals marked with the number 3. The same type of signals recorded in the last stage of cracking the sample tensile steel S355JR, when there was no cracking at the brittle mechanisms (Figure 6). Signals marked in blue and number 1 come mainly from elastic and plastic deformations, as well as from individual noise generated by the strength machine and the loading system. These signals, therefore, probably can be equated with the complex, rapid dislocation motion process in ferrite and the act of fracture.

**Figure 7:**
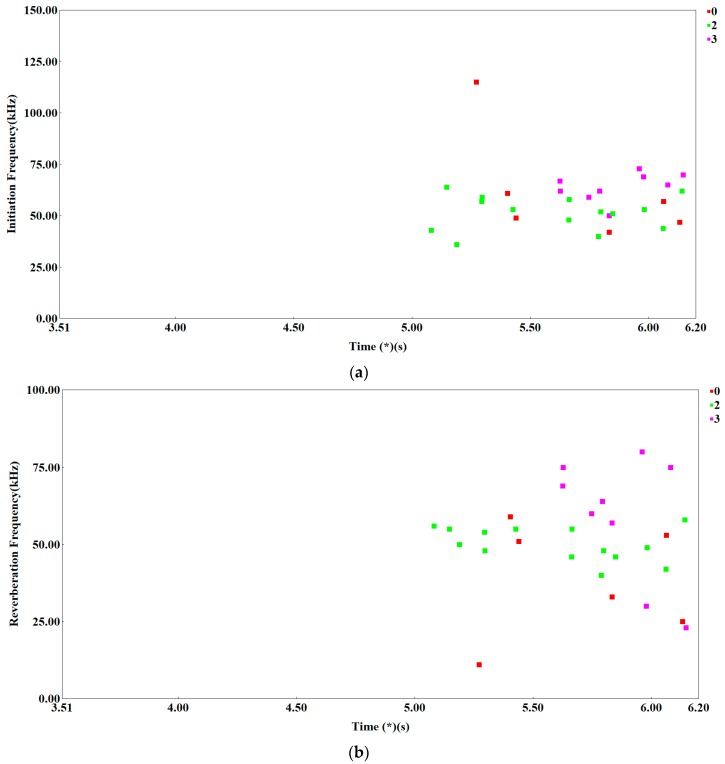
Scatterplot: (**a**) Initiation frequency (IF) (kHz) vs. time (s); and (**b**) reverberation frequency (RF) (kHz) vs. time (s).

Figure 7a,b presents the parameters of the acoustic emission signal: initiation frequency (IF) (kHz) and reverberation frequency (RF) (kHz) vs. time and signal classes. Analysing these graphs, it can be seen that green signals have a similar frequency range in the initial stage (IF) parameter as well as in the later stage (RF) parameter during signal duration. This range falls within the frequency range of pink signals.

We can, therefore, assume that the signals of green color characterize the process of dislocation motion, and cracking on the cleavage surfaces in ferrite, i.e., brittle cleavage fracture. The red signals show different frequencies at different stages of the load frequency band. Parameter (IF) signals occurring in the earlier stage of the load have much higher than others, which allows to assume that these signals are generated in a different environment than ferrite. These may be signals from the cracking of precipitates segregated at the ferrite grain boundaries or connected with the process of de-bonding from the ferrite matrix. These signals can be imitated, in ferrite, and in precipitations. That is why there is a different frequency in parameter (IF).

The signal imitated in the precipitations should be short. However, the signal imitated in ferrite is longer and therefore parameter (RF) is similar. In the next stage of loading, red signals characterize the development of intercrystallite fracture growth and are imitated in ferrite.

## 4. Conclusions

### 4.1. General Conclusions

To raise AE technology to the next level, we must improve it from several fronts. Only then can AE become a serious player in structural integrity assessment. We need to better characterize AE sources; to extract more from AE signals reaching sensors; to devise effective AE parameters; to integrate new analysis methods, such as wave transformation analysis(WT), moment tensor analysis (MTA), and pattern recognition analysis (PRA); to include simulation tools in analyses; to accumulate basic data on structures with standardized procedures; and to devise combinatorial approach between localized damage evaluation and long-range detection and to develop regional or global database under international cooperation. Using systematic approach along with NDT, fracture mechanics, etc., improved AE can offer more value to industrial users and to contribute substantially to structural integrity assessment. Development of the proposed AE monitoring technique reported in this paper facilitates for the prognostics and life predictions of the structure. The developed methodology can be utilized for continuous in-service monitoring of structures and has proven to be promising for use in practice.

### 4.2. Specific Applications

1. Application of the k-means grouping method based on the analysis in the 13 parameter space of AE signals provides positive results in the classification of destructive processes in fracture toughness K_Ic_ tests.
2. Application of the k-means grouping method allows differentiating between brittle fractures mechanisms.
3. The process of brittle fracture was shown to be a sequential process composed of inter-grain cracking, brittle-cleavage and ductile fracture.
4. Acoustic emission technology was found to be able to successfully monitor and detect real-time crack initiation and also separate and identify AE signals that correspond to different fracture mechanisms
5. The grouping analysis shows that some AE parameters noticeably change after the initiation of the crack (absolute energy, initial frequency, reverberation frequency), which indicates their high independence and suitability for monitoring the cracking process.
6. The developed base of reference signals forms the theoretical basis for using pattern-based AE technology to detect and monitor crack propagation in structures that do not have a “load history” to assess the safety of steel structures using the principles of fracture mechanics.

## References

[1] Koçak M., Webster S., Janosch J.J., Ainsworth R.A., Koerc R., FITNET: Fitness-for-Service (2008). Fracture-Fatigue-Creep-Corrosion.

[2] Ono K. (2011). Application of Acoustic Emission for Structure Diagnosis. Diagn.-Diagn. Struct. Health Monit..

[3] Adamczak A., Świt G., Krampikowska A. (2019). Application of the Acoustic Emission Method in the Assessment of the Technical Condition of Steel Structures. IOP Conf. Ser. Mat. Sci. Eng..

[4] Świt G., Krampikowska A. Influence of the number of acoustic emission descriptors on the accuracy of destructive process identification in concrete structures. Proceedings of the 7th IEEE Prognostics and System Health Management Conference (PHM-Chengdu).

[5] Świt G. (2018). Acoustic emission method for locating and identifying active destructive processes in operating facilities. Appl. Sci..

[6] ASTM E647 (2010). Standard Test Method for Measurement of Fatigue Crack Growth Rates.

[7] Biancolini M.E., Brutti C., Paparo G., Zanini A. (2006). Fatigue cracks nucleation on steel, Acoustic emission and fractal analysis. Int. J. Fatigue.

[8] Caesarendra W., Kosasih B., Tieu A.K., Zhu H., Moodie C.A.S., Zhu Q. (2016). Acoustic emission-based condition monitoring methods: Review and application for low speed slew bearing. Mech. Syst. Signal Process..

[9] Keshtgar A., Modarres M. (2013). Detecting Crack Initiation Based on Acoustic Emission. Chem. Eng. Trans..

[10] Rabiei M., Modarres M. (2013). Quantitative methods for structural health management using in situ acoustic emission monitoring. Int. J. Fatigue.

[11] Zhang X., Feng N., Wang Y., Shen Y. (2015). Acoustic emission detection of rail defect based on wavelet transform and Shannon entropy. J. Sound Vib..

[12] Keshtgar A., Modarres M. Acoustic Emission-Based Fatigue Crack Growth Prediction. Proceedings of the Reliability and Maintainability Symposium (RAMS).

[13] Yan Z., Chen B., Tian H., Cheng X., Yang J. (2015). Acoustic detection of cracks in the anvil of a large-volume cubic high-pressure apparatus. Rev. Sci. Instrum..

[14] Gao L.X., Zai F.L., Su S.B., Wang H.Q., Chen P., Liu L.M. (2011). Study and application of acoustic emission testing in fault diagnosis of low-speed heavy-duty gears. Sensors.

[15] Qu Y.Z., He D., Yoon J., Van Hecke B., Bechhoefer E., Zhu J.D. (2014). Gearbox tooth cut fault diagnostics using acoustic emission and vibration sensors—A comparative study. Sensors.

[16] Doan D.D., Ramasso E., Placet V., Zhang S., Boubakar L., Zerhouni N. (2015). An unsupervised pattern recognition approach for AE data originating from fatigue tests on polymer-composite materials. Mech. Syst. Signal Process..

[17] Ting-Hua Y., Stathis C., Stiros X.-W.Y., Jun L. (2014). Structural Health Monitoring—Oriented Data Mining, Feature Extraction, and Condition Assessment.

[18] Tang J., Soua S., Mares C., Gan T.-H. (2016). An experimental study of acoustic emission methodology for in service condition monitoring of wind turbine blades. Renew. Energy.

[19] The MathWorks, Inc. (2017). Statistics and Machine Learning Toolbox.

[20] Li L., Lomov S.V., Yan X., Carvelli V. (2014). Cluster analysis of acoustic emission signals for 2D and 3D woven glass/epoxy composites. Compos. Struct..

[21] Crivelli D., Guagliano M., Monici A. (2014). Development of an artificial neural network processing technique for the analysis of damage evolution in pultruded composites with acoustic emission. Compos. Part B.

[22] Desgraupes B. (2013). Clustering Indices.

[23] Eftekharnejad B., Mba D. (2011). Monitoring natural pitting progress on helical gear mesh using acoustic emission and vibration. Strain.

[24] Zhu X., Zhong C., Zhe J. (2017). A high sensitivity wear debris sensor using ferrite cores for online oil condition monitoring. Meas. Sci. Technol..

[25] Li R., He D. (2012). Rotational machine health monitoring and fault detection using EMD—Based acoustic emission feature quantification. IEEE Trans. Instrum. Meas..

[26] Loutas T.H., Roulias D., Pauly E., Kostopoulos V. (2011). The combined use of vibration, acoustic emission and oil debris on-line monitoring towards a more effective condition monitoring of rotating machinery. Mech. Syst. Signal Process..

[27] Hase A., Mishina H., Wada M. (2012). Correlation between features of acoustic emission signals and mechanical wear mechanisms. Wear.

[28] Li R., Seçkiner S.U., He D., Bechhoefer E., Menon P. (2012). Gear fault location detection for split torque gearbox using AE sensors. IEEE Trans. Syst. Man Cybern. Part C Appl. Rev..

[29] Gu D., Kim J., An Y., Choi B. (2011). Detection of faults in gearboxes using acoustic emission signal. J. Mech. Sci. Technol..

[30] Zhang L., Yalcinkaya H., Ozevin D. (2017). Numerical Approach to Absolute Calibration of Piezoelectric Acoustic Emission Sensors using Multiphysics Simulations. Sens. Actuators A Phys..

[31] Zhang L., Ozevin D., Hardman W., Timmons A. (2017). Acoustic emission signatures of fatigue damage in idealized bevel gear spline for localized sensing. Metals.

[32] ASTM E8 (2003). Standard Test Method for Tension Testing of Metallic Materials.

[33] ASTM E1820-09 (2011). Standard Test. Method for Measurement of Fracture Toughness.

[34] ISO 12135:2002 (2002). Metallic Materials—Unified Method of Test for the Determination of Quasistatic Fracture Toughness.

[35] Schwalbe K.H., Landes J.D., Heerens J. (2007). Classical Fracture Mechanics Methods.

[36] Anderson T.L. (2008). Fracture Mechanics: Fundamentals and Applications.

[37] Neimitz A. (1999). Mechanika Pękania.

[38] Pała R., Dzioba I. (2018). Influence of delamination on the parameters of triaxial state of stress before the front of the main crack. AIP Conf. Proc..

